# Facial expression (mood) recognition from facial images using committee neural networks

**DOI:** 10.1186/1475-925X-8-16

**Published:** 2009-08-05

**Authors:** Saket S Kulkarni, Narender P Reddy, SI Hariharan

**Affiliations:** 1 Department of Biomedical Engineering, University of Akron, Akron, OH 44325-0302; USA; 2 Department of Electrical and Computer Engineering, University of Akron, Akron, OH 44325-3904, USA

## Abstract

**Background:**

Facial expressions are important in facilitating human communication and interactions. Also, they are used as an important tool in behavioural studies and in medical rehabilitation. Facial image based mood detection techniques may provide a fast and practical approach for non-invasive mood detection. The purpose of the present study was to develop an intelligent system for facial image based expression classification using committee neural networks.

**Methods:**

Several facial parameters were extracted from a facial image and were used to train several generalized and specialized neural networks. Based on initial testing, the best performing generalized and specialized neural networks were recruited into decision making committees which formed an integrated committee neural network system. The integrated committee neural network system was then evaluated using data obtained from subjects not used in training or in initial testing.

**Results and conclusion:**

The system correctly identified the correct facial expression in 255 of the 282 images (90.43% of the cases), from 62 subjects not used in training or in initial testing. Committee neural networks offer a potential tool for image based mood detection.

## Background

Facial expressions and related changes in facial patterns give us information about the emotional state of the person and help to regulate conversations with the person. Moreover, these expressions help in understanding the overall mood of the person in a better way. Facial expressions play an important role in human interactions and non-verbal communication. Classification of facial expressions could be used as an effective tool in behavioural studies and in medical rehabilitation. Facial expression analysis deals with visually recognizing and analyzing different facial motions and facial feature changes. Ekman and Friesen [1] developed the facial action coding system (FACS) to measure the facial behaviour. The FACS codes different facial movements into Action Units (AU) based on the underlying muscular activity that produces momentary changes in the facial expression. An expression is further recognized by correctly identifying the action unit or combination of action units related to a particular expression.

Numerous investigators [2-10] have used neural networks for facial expression classification. The performance of a neural network depends on several factors including the initial random weights, the training data, the activation function used, and the structure of the network including the number of hidden layer neurons, etc. Reddy and Buch [11], Das et al [12], Gopinath and Reddy [13], Srirao et al. [14] and Reddy et al. [15] developed the concept of committee neural networks in which a large number of networks are trained. Based on initial testing with data obtained from subjects not used in training, a few networks (e.g. 5) are recruited into a committee. Final evaluation of the committee is conducted with data obtained form subjects not used in training or in initial testing. Each member of the committee then classifies the image. The decision output of the member networks is fused by majority voting. These authors observed that a committee neural network system provides an improved performance when compared to a single network. The question remains if a committee or committees of neural networks trained on back-propagation can provide a reasonable (close to 90%) accuracy in classification of different facial expressions. The purpose of the present research was to address this question by developing and evaluating a committee neural network classification system to classify facial expressions (moods) using static facial images.

## Methods

The database used in the study consisted of facial expression images from the Cohn-Kanade database [16]. Two types of parameters were extracted from the facial image: real valued and binary. A total of 15 parameters consisting of eight real-valued parameters and seven binary parameters were extracted from each facial image. The real valued parameters were normalized. Generalized neural networks were trained with all fifteen parameters as inputs. There were seven output nodes corresponding to the seven facial expressions (neutral, angry, disgust, fear, happy, sad and surprised).

**Figure 1:**
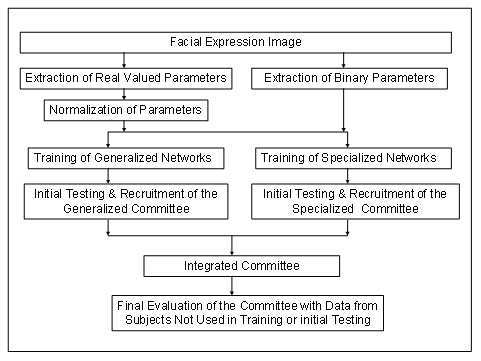
**An overall block diagram of the methodology**.

Based on initial testing, the best performing neural networks were recruited to form a generalized committee for expression classification. Due to a number of ambiguous and no-classification cases during the initial testing, specialized neural networks were trained for angry, disgust, fear and sad expression. Then, the best performing neural networks were recruited into a specialized committee to perform specialized classification. A final integrated committee neural network classification system was built utilizing both generalized committee networks and specialized committee networks. Then, the integrated committee neural network classification system was evaluated with an independent expression dataset not used in training or in initial testing. A generalized block diagram of the entire system is shown in Figure 1.

### Facial Image Database

**Figure 2:**
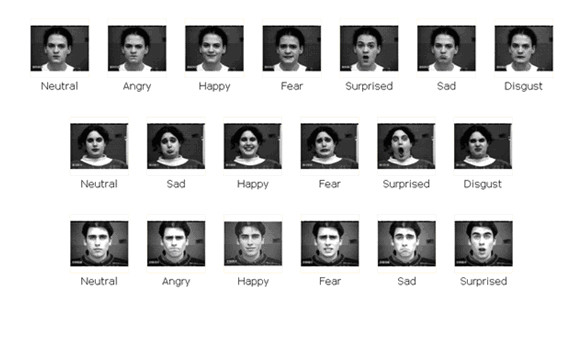
**Facial images in different expressions from the Cohn: Kanade database (reproduced with permission from Cohn-Kanade database **[16]**)**

Facial expression images were obtained from the Cohn-Kanade [16] database. The database contained facial images taken from 97 subjects with age ranging from 18 to 30 years. The database had 65 percent female subjects. Fifteen percent of the subjects were African-American and three percent were Asian or Latino. The database images were taken with a Panasonic camera (model WV 3230). The camera was located directly in front of the subject. The subjects performed different facial displays (single action units and combinations of action units) starting and ending with a neutral face. The displays were based on descriptions of prototypic emotions (i.e., neutral, happy, surprise, anger, fear, disgust, and sad). The image sequences were digitized into 640 by 480 pixel arrays with 8-bit precision for gray scale values. Figure 2 gives examples of various expressions by different subjects.

Although the database contained 2000 images, many images were repetitions (frames of same subjects in same moods): hence, the entire dataset was not used for the study. In fact, using repetitions would increase the accuracy, but essentially would be analyzing somewhat similar expressions of the same subject. The purpose of the study was not to test the response of the classification engine on repetitive images, but was to test it on a variety of images. Thus, in order to study the robustness of the system for different subject-mood variations, selection of images for this study was based on selecting a unique combination of subject-mood. The present study utilized 467 images from 97 subjects.

### Image Processing and Feature Extraction

Two types of parameters were extracted from the facial images of 97 subjects: (1) real valued parameters and (2) binary parameters. The real valued parameters have a definite value depending upon the distance measured. This definite value was measured in number of pixels. The binary measures gave either a present (= 1) or an absent (= 0) value. In all, eight real valued measures and seven binary measures were obtained.

A number of parameters, both real-valued and binary, were extracted and analyzed to decide their effectiveness in identifying a certain facial expression. The features which did not provide any effective information of the facial expression portrayed in the image were eliminated and were not used in the final study. The real valued and binary feature selection was inspired by the FACS. The following real valued and binary parameters were finally used in the study.

### Real valued parameters

1. ***Eyebrow raise distance ***– The distance between the junction point of the upper and the lower eyelid and the lower central tip of the eyebrow.

2. ***Upper eyelid to eyebrow distance ***– The distance between the upper eyelid and eyebrow surface.

3. ***Inter-eyebrow distance ***– The distance between the lower central tips of both the eyebrows.

4. ***Upper eyelid *– *lower eyelid distance ***– The distance between the upper eyelid and lower eyelid.

5. ***Top lip thickness ***– The measure of the thickness of the top lip.

6. ***Lower lip thickness ***– The measure of the thickness of the lower lip.

7. ***Mouth width ***– The distance between the tips of the lip corner.

8. ***Mouth opening ***– The distance between the lower surface of top lip and upper surface of lower lip.

**Figure 3:**
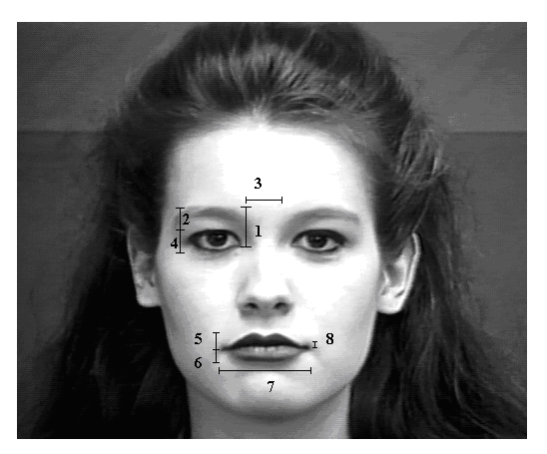
**Real-valued measures from a sample neutral expression image**. 1-eyebrow raise distance, 2-upper eyelid to eyebrow distance, 3-inter eyebrow distance, 4-upper eyelid to lower eyelid distance, 5-top lip thickness, 6-lower lip thickness, 7-mouth width, 8-mouth opening. (Facial expression image from the Cohn-Kanade database [16]. Used with permission)

The real valued parameters are depicted in Figure 3.

### Binary parameters

1. ***Upper teeth visible ***– Presence or absence of visibility of upper teeth.

2. ***Lower teeth visible ***– Presence or absence of visibility of lower teeth.

3. ***Forehead lines ***– Presence or absence of wrinkles in the upper part of the forehead.

4. ***Eyebrow lines ***– Presence or absence of wrinkles in the region above the eyebrows.

5. ***Nose lines ***– Presence or absence of wrinkles in the region between the eyebrows extending over the nose.

6. ***Chin lines ***– Presence or absence of wrinkles or lines on the chin region just below the lower lip.

7. ***Nasolabial lines ***– Presence or absence of thick lines on both sides of the nose extending down to the upper lip.

**Figure 4:**
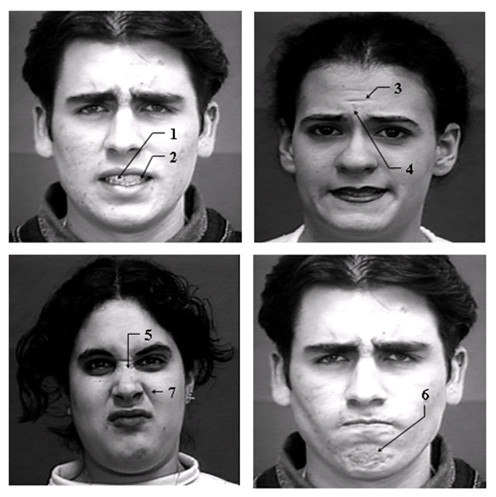
**Binary measures from sample expression images**. 1-upper teeth visible, 2-lower teeth visible, 3-forehead lines, 4-eyebrow lines, 5-nose lines, 6-chin lines, 7-nasolabial lines. (Facial expression image from the Cohn-Kanade database [16]. Used with permission).

These binary parameters are depicted in Figure 4

The real valued parameters were the distances (in number of pixels) measured between specified facial features. In case of parameters involving features which were symmetrically present on both sides of the face, an average of both the measurements was obtained. Real-valued measures were obtained for expressions including the neutral image. The real valued parameters were then normalized in the following manner:



All the parameters were extracted by manual and/or semi-automatic techniques. The purpose of the present study was to evaluate the efficacy of committee neural networks. Therefore, no effort was made to develop automated techniques for feature extraction.

The binary parameters were characterized by the presence or absence of the facial muscle contractions or the facial patterns formed due to these contractions. An edge detection algorithm was applied to the image to determine if the pattern was present or absent. A simple canny edge detector (MATLAB based) was used to determine whether a pattern of lines existed which further decided the binary feature was true (1) or false (0).

The eight normalized real valued parameters together with the seven binary parameters were fed to neural networks. The entire dataset from 97 subjects (467 images) was divided into three groups: 25 subjects (139 images) for training, 10 subjects (46 images) for initial testing, and 62 subjects (282 images) for final evaluation.

### Training of generalized neural networks

Several multi layered, fully connected, feed forward neural networks were trained to classify different expressions. A total of 105 networks were trained using different number of hidden layers (2, 3, 4, 5), different initial weights, different number of neurons in the hidden layers (7, 14, 15, 28, 45, 60), and different transfer functions.

Each network had fifteen input nodes, each corresponding to the fifteen input parameters. Each of these networks had seven output nodes, each corresponding to one of the seven expressions (neutral, angry, disgust, fear, happy, sad and surprised). Since the normalized input data was in the range of -1 to 1, the "tansig" function was used for the hidden layer neurons. The output of the neural network has to be in the 0 to 1 range. Thus, the "logsig" function was used as the transfer function for the output layer neurons. The output of each node was converted to a binary number (either 0 or 1). An output of 0.6 or more was forced to 1 and an output of less than 0.6 was forced to 0. An output of 1 indicated that particular expression was present and output of 0 indicated that particular expression was absent. We have varied the threshold from 0.55 to 0.9 and found that a threshold of 0.6 gave better results.

The networks were trained using the Levenberg-Marquardt (trainlm, a modified back propagation) technique using MATLAB. The error goal was set at 1*10^-10 ^and the maximum number of epochs used for training was varied from 100–1000.

### Recruitment of the generalized committee neural networks

**Figure 5:**
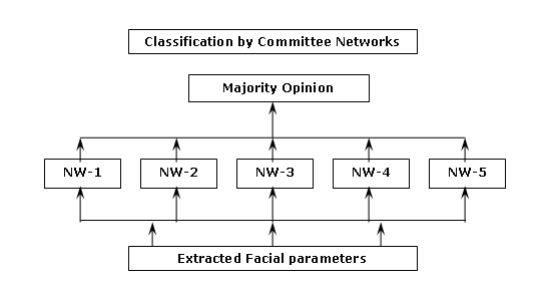
**Five-network committee neural network architecture**.

All of the 105 trained neural networks were subject to initial testing using data from ten subjects (46 datasets) not used in training. The best performing networks were recruited into a committee. Committees of sizes 3, 5, 7, 9, 11 and 13 networks were formed and evaluated with the same initial testing data. The 11 member committee provided the best performance in the initial evaluation. Figure 5 shows the block diagram of such a five network committee architecture.

### Training of Specialized neural networks

The initial evaluation of the committee classification system presented some all-zero or no-classification cases. These no-classification cases resulted when the input data was from the angry, disgust, fear or sad expressions. Twenty specialized networks were trained to perform classification of these four (angry, disgust, fear and sad) expressions with an aim to reduce the number of no-classification cases. These networks also had binary outputs at each output node. Training data for the specialized networks were extracted from the same 25 subjects used for training the generalized networks

### Recruitment of specialized committee of networks

All of the 20-specialized neural networks were subject to initial testing using data from ten subjects not used in the training. From this, three networks were recruited to form the specialized committee of neural networks.

### Evaluation of the integrated committee neural network system

**Figure 6:**
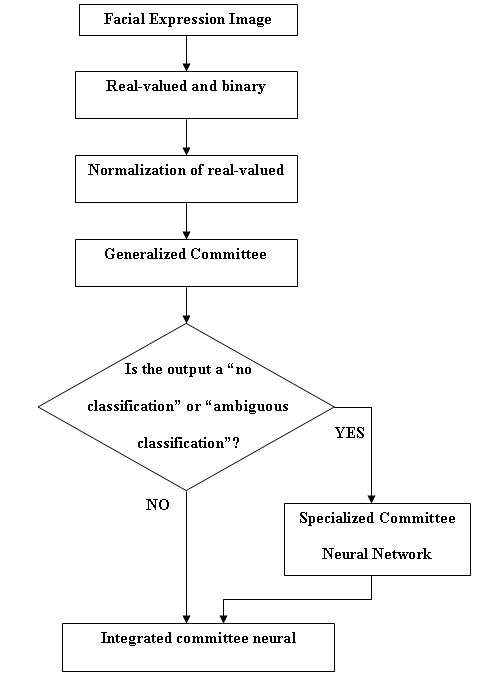
**A flowchart of the overall classification system**.

An integrated committee neural network system was formed incorporating the eleven member generalized committee and three member specialized committee. Figure 6 shows the flowchart of the integrated committee neural network system classification process. Data from 62 subjects was used for final evaluation of the integrated system. These sixty-two subjects were independent subjects not used in training or in initial evaluations. Input data was first fed to the generalized committee neural network classification system. If the output of the generalized classification system was no classification (all zeros) or ambiguous (more than one expression), then, the same input data was fed to the specialized networks. The specialized networks further classified the expression into angry, disgust, fear or sad. Finally, the generalized and the specialized committee network outputs were combined to present the final expression classification system.

## Results

Real valued and binary parameters were extracted from the facial images from 97 subjects (467 images). Table 1 shows the average percentage deviations from the corresponding neutral values, for all of the eight real valued parameters. Table 2 shows the average percentage presence of the seven binary parameters for all of the seven expressions.

**Table 1:** Average percentage deviation of the eight real valued parameters for different expressions from 97 subjects

***Real valued parameter***	**Average percentage deviation from neutral value**						
	*Neutral*	*Angry*	*Disgust*	*Fear*	*Happy*	*Sad*	*Surprised*

*Eyebrow raise* *distance*	0.00	-28.04	-36.86	1.43	1.54	5.29	49.67

*Upper eyelid-* *eyebrow distance*	0.00	-31.18	-26.47	4.15	11.91	15.03	106.12

*Inter eyebrow* *distance*	0.00	-22.36	-9.45	-16.09	-0.50	-6.43	5.62

*Upper eyelid-* *lower eyelid* *distance*	0.00	-34.60	-49.52	-18.69	-20.07	-13.04	25.13

*Top lip* *thickness*	0.00	-49.99	-10.20	-17.46	-11.04	-23.31	1.27

*Bottom lip* *thickness*	0.00	-51.77	-6.83	-23.55	-10.92	-8.80	23.65

*Mouth width*	0.00	-8.19	-7.48	9.90	33.35	-4.04	-14.62

*Mouth opening*	0.00	-7.74	95.37	738.30	785.81	2.55	2489.00

**Table 2:** Average percentage presence of the seven binary parameters for different expressions

***Binary parameter***	**Average percentage presence of parameter**						
	*Neutral*	*Angry*	*Disgust*	*Fear*	*Happy*	*Sad*	*Surprised*

*Upper teeth visible*	0.00	0.00	13.33	93.10	83.91	0.00	57.89

*Lower teeth visible*	0.00	0.00	6.67	77.59	37.93	0.00	47.37

*Forehead lines*	0.00	0.00	2.22	29.31	0.00	14.52	69.74

*Eyebrow lines*	0.00	73.81	86.67	50.00	0.00	20.97	30.26

*Nose lines*	0.00	61.90	88.89	31.03	0.00	4.84	0.00

*Chin lines*	0.00	80.95	22.22	10.34	0.00	85.48	0.00

*Nasolabial lines*	0.00	16.67	84.44	56.90	97.70	9.68	9.21

Averages were calculated over the entire dataset of ninety-seven subjects

The integrated committee classification system correctly identified 255 out of 282 different expressions from sixty-two different subjects. There were 27 incorrect classifications. The incorrect classifications were either misclassifications, ambiguous classification or no-classification cases. A misclassification occured when an expression was not accurately categorized. An ambiguous classification occured when two or more expressions were identified for a classification output. A no-classification occured when there was an all zero output and no expression was classified.

**Figure 7:**
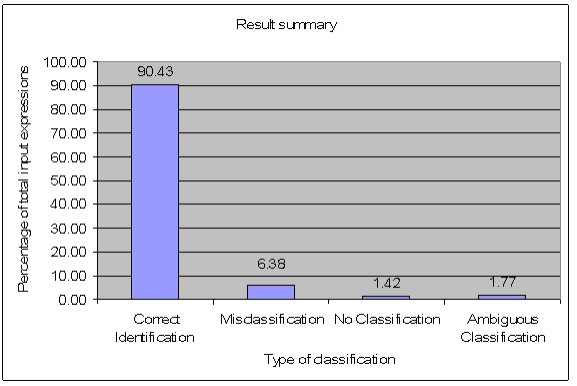
**Plot of percentage of total input expressions versus type of classification**.

**Figure 8:**
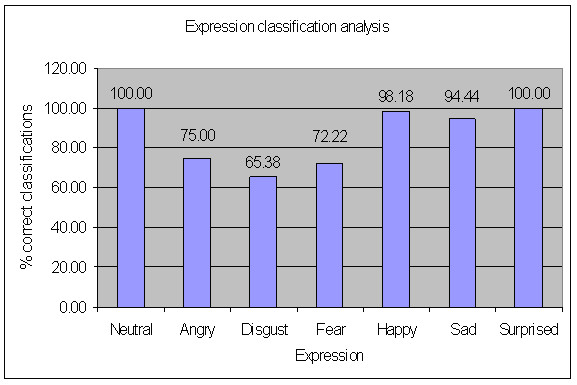
**Plot of percentage correct classifications for different expressions**.

There were eighteen misclassification cases, four no classification cases and five ambiguous classification cases amongst the 282 expressions evaluated. Table 3 shows the confusion matrix. The matrix shows the system classification versus the actual expression presented. For instance, when 55 happy expressions were presented, the integrated committee classified 54 expressions as happy and classified one expression as a combination of happy and fear. When 36 fear expressions were presented, the committee correctly classified 26 cases as fear. It classified one expression as disgust, seven expressions as happy and two expressions as a combination of fear and disgust. Figure 7 shows a comparative graphical summary of the integrated committee neural network system performance. Figure 8 gives a plot of expression wise performance of the integrated committee neural network system. The angry, disgust and fear expressions showed low classification accuracy (in the range of 65% to 75%) while the happy, sad and surprised expressions showed high classification accuracy (more than 90%). Table 4 presents the number of correct classifications by the individual networks and the committee network.

**Table 3:** The Confusion Matrix

***Expressions presented***	***System Classification***											***Total***
	**N**	**A**	**D**	**F**	**H**	**S**	**Su**	**Z**	**AD**	**DF**	**FH**	
	
**Neutral (**N**)**	62											62

**Angry (A)**		15	1			3		1				20

**Disgust (D)**		2	17	2		1		2	2			26

**Fear (F)**			1	26	7					2		36

**Happy (H)**					54						1	55

**Sad (S)**		1				34		1				36

**Surprised (**Su**)**							47					47

**Total**	62	18	19	28	61	38	47	4	2	2	1	282

The first column represents the expression tested. The last column of each row represents the total number of images of the corresponding expression tested (e.g. Out of 20 angry expressions tested 15 were classified as angry, 1 as disgust, 3 as sad, and one had no classification (all zero output). 2 disgust cases were classified as angry and 1 sad case was classified as angry. LEGEND: N: Neutral; A: Angry; D: Disgust,; F: Fear; H: Happy; S: Sad; Su: Surprised; Z: All Zero (no classification); AD: Angry-Disgust; DF – Disgust-Fear; FH: Fear-Happy

**Table 4:** Number of correct classifications by individual networks and by the committee

**Neural Networks**	**Designated expression correctly** **identified out of 282 expressions** **presented**.
NN-1	198

NN-2	182

NN-3	194

NN-4	191

NN-5	204

NN-6	206

NN-7	204

NN-8	213

NN-9	204

NN-10	217

NN-11	203

**Integrated** **Committee Results**	**255**

## Discussion

The present study demonstrated the development and the application of committee neural networks to classify seven basic emotion types from facial images. The integrated committee neural network system consisting of generalized and specialized networks, can classify the emotion depicted in the facial image into one of the following emotions: neutral, angry, disgust, fear, sad, surprised or happy. The integrated committee decision provided accurate and reliable classification in 90.43% of the 282 cases from 62 subjects evaluated (Table 3).

The database used for the expression analysis consisted of subjects who performed a series of different expressions. The variability and reliability of these expressions introduced different levels in the same expression. This introduced variability in the overall dataset. In addition, the database consisted of mostly expressions of a deliberate nature. There is a significant difference between expressions of a spontaneous and of a deliberate nature. Unless the database consists of both spontaneous and deliberate expressions, the expression analysis system cannot be robust enough to detect the exact expression correctly.

Further variability is introduced because the expression databases are classified only into six basic facial expression types (angry, disgust, fear, happy, sad and surprised). In reality, an expression is often a combination of two or more of the prototypic expressions, Also, expressions are assumed to be singular and to begin and end with a neutral position. In reality, facial expressions are much more complex and occur in different combinations and intensities. Therefore, an identified expression could be a combination of two different expressions with one of them being more dominant in intensity. The classifier, therefore, should be smart enough to correctly identify the combination of expressions and each expression's individual intensity. In the present study, five expressions were classified as having a combination of the correct expression and some other expression (Table 3).

The performance of a neural network depends on the type of parameters extracted from the facial image. The performance also depends on the processing of the parameter data before presentation to the networks. Pantic and Rothkrantz [17] have developed a model based on 25 features and 19 facial points based on the frontal images of the face and 10 points based on the profile image of the face. Kobayashi and Hara [2,18,19] have developed a geometric face model based on 30 feature characteristic points. The seven real valued and eight binary parameters used in the present study gave an equal or a slightly better recognition rate than most other methods such as feature point tracking, Gabor wavelet analysis [10] and optical flow tracking.

Real valued parameters displayed negative deviation, positive deviation or no substantial deviation from the neutral value (Table 1). The trend of variation of different parameters with respect to neutral values for different expressions helps in the effective training of neural networks to recognize specific expressions. Together the real valued and binary parameters (Table 2) characterize each expression. However, some parameters do not display substantial deviation from neutral value for certain expressions and hence, do not contribute in recognizing that particular expression.

In the present study, the committee neural network system performed better than an individual network (Table 4). No single network classification results were as good as committee classification results.

Each neural network had a single output node for each expression. The output of each output node is binary (present or absent). For the individual member network classification, one approach is to use a "winner takes all" and have each member of the committee produce only one output. This process produces good results. However, for numerous biomedical applications, due to significant biological variability, such an approach can produce misclassifications, if the network is presented with data from entirely new subjects with extreme features. This is especially the case if the winner node has an output which is not more than 10% larger than another node. Therefore, our approach is to let a network produce more than one classification. For example, a patient simultaneously can have disease A and disease B. Our technique is to take the output of each output node of a network and compare it with a threshold, and if the output exceeds the threshold, then the output is made equal to one, otherwise it is set equal to zero. Even though this approach can yield multiple classifications (ambiguous classification) or no-classification, our previous studies [11-15] have shown this technique to yield better results. Therefore, we have taken this approach in the present study.

In the present study, logistic sigmoid units were used as the output transfer functions, and tangential sigmoid functions were used for the middle layer neurons. Zhang et al. [4] used the softmax function, which gave a probability distribution. The recognition rate increased with increasing number of nodes (neurons) in the hidden layer. The recognition rate was 90.1% with 7 hidden nodes. However, when they excluded the fear expression, they achieved a recognition rate of 92.2% with 7 hidden nodes. The softmax function essentially provides a normalized output by dividing the actual nodal output with the sum of all nodal outputs in the output layer. In the present study, for the ambiguous cases, the probabilities (softmax function of the outputs) were close to each other (e.g. outputs of 0.69, 0.71 with probabilities of 0.493 and 0.507). We have decided to convert the outputs into a binary format.

In the present study, two committees were developed. One committee classified the image into one of the seven classes. If the majority of the networks in the first committee provide a zero output (less than threshold) or an ambiguous (multiple classifications, i.e. more than one output of 1), then, we set the first committee output as "no-classification or ambiguous classification" and sent it to the second committee. We called the first committee as the generalized committee and the second committee as the specialized committee. This is similar to first visiting a general physician followed by a referral to a specialized physician if needed.

The integrated committee neural network classification system, consisting of a combination of generalized committee networks and specialized committee networks, performed well (Table 3, Figure 7). It was observed that the angry, disgust, sad and fear expressions were difficult to classify (Figure 8). These four expressions are negative emotions which are often difficult to classify. They also often occur in combinations, with one of them having higher intensity than the other. All of the incorrect classifications observed in the present study involved one of these four expressions (Table 3).

Fasel and Luettin [8] and Pantic and Rothkrantz [17] have summarized the results for various face based emotion classification systems reported in the literature. Table 5 presents a summary of results reported in the literature [5,7,9,10,17,18,20-23]. The average expression recognition rate of all of these systems is around 88%. (75% to 100%). Some of these studies have used either limited testing data or the same data both for training and for testing. In comparison, the integrated committee neural network system developed in the present study was trained with 139 images and tested with 282 images drawn from subjects not used in training. The classification system in the present study yielded 90.43% correct classifications. In reality, the system was tested with a total of 328 images including 46 images used for initial testing. The accuracy would be even higher if the initial testing results were to be included in the overall results. Although the five ambiguous classifications were considered as incorrect classifications, it can be observed from Table 3 that all of these five cases gave an additional classification in addition to the correct classification (e.g. fear and happy instead of fear, angry and disgust instead of angry).

**Table 5:** Testing Reported in the Literature

**Authors**	**Subjects** **tested**	**No. images tested**	**Percentage** **accuracy**
Edwards [20]	25	200	74

Kobayashi & Hara [18]	15	90	85

Pantic & othkrantz [17]	8	256	91

Lyons et al [21]	10	193	92 75 (with new subjects)

Huang & Huang [22]	15	90	75

Hong et al [23]	25	175	81

Zhang [7,9]	10	213	90.1

Zhao & kearney [5]		94	100

Sebe et al. [10]			88 to 95

Present study	62	282	90.4

Perhaps in the future, the accuracy could be improved further by first classifying the image into a neutral, positive (happy and surprised) and negative (angry, disgust, fear, and sad) mood. Then, the image could be sub-classified by utilizing specialized committee networks. Also, the parameters which play an important role could be identified.

## Conclusion

Eight real valued and seven binary parameters were successfully extracted from 97 subjects (467 facial images) for seven different expressions (neutral, angry, disgust, fear, sad, happy and surprised). An integrated committee neural network system was developed incorporating a generalized neural network committee and a specialized neural network committee.

Several (105) generalized neural networks (with different initial weights, structure, etc) were trained to classify the image into seven different expressions (neutral, angry, disgust, fear, sad, happy and surprised). Similarly, several (20) specialized neural networks were trained to classify the image into four different expressions (angry, disgust, fear and sad). All of the networks were tested with initial testing data derived from subjects not used in training. The best performing networks were recruited into a generalized committee and a specialized committee. If the generalized committee gave an ambiguous output or no-classification, then, the data was fed to a specialized committee. The integrated committee system was evaluated with data not used in training or in initial testing.

The integrated system correctly classified the expressions in 255 cases out of 282 cases (90.43%) from 62 subjects. There were 18 misclassifications, 4 no-classifications and 5 cases of ambiguous classifications (combination of another expression in addition to the correct expression). No single network performed as good as the committee network. Committee neural network based intelligent systems offer a useful tool for image based expression classification.

## Abbreviations

FACS: Facial Action Coding System; AU: Action Units; JAFFE: Japanese Female Facial Expression; PCA: Principal Components Analysis.

## Competing interests

The authors declare that they have no competing interests.

## Authors' contributions

All authors have read and approved the final manuscript. SSK participated in planning, image analysis, parameter extraction, data processing, neural network training, development of committee neural networks and data interpretation. NPR conceived the project and participated in planning of the project, committee neural network development and data interpretation. SIH participated in data interpretation.
